# *Ficus pandurata* Hance Inhibits Ulcerative Colitis and Colitis-Associated Secondary Liver Damage of Mice by Enhancing Antioxidation Activity

**DOI:** 10.1155/2021/2617881

**Published:** 2021-12-18

**Authors:** Weibo Dai, Xinyi Zhan, Weijie Peng, Xin Liu, Weiwen Peng, Quanxi Mei, Xianjing Hu

**Affiliations:** ^1^Pharmacology Laboratory, Zhongshan Hospital, Guangzhou University of Chinese Medicine, Zhongshan, 528401 Guangdong, China; ^2^Shenzhen Baoan Authentic TCM Therapy Hospital, Shenzhen, 518101 Guangdong, China; ^3^Centre for Cancer and Inflammation Research, School of Chinese Medicine, Hong Kong Baptist University, Hong Kong SAR, China; ^4^Biotechnological Institute of Chinese Materia Medica, Jinan University, Guangzhou, 510632 Guangdong, China

## Abstract

Inflammatory bowel disease (IBD), a global disease threatening human health, is commonly accompanied by secondary liver damage (SLD) mediated by the gut-liver axis. Oxidative stress acts a critical role in the onset of IBD, during which excessive oxidation would destroy the tight junctions between intestinal cells, promote proinflammatory factors to penetrate, and thereby damage the intestinal mucosa. *Ficus pandurata* Hance (FPH) is widely used for daily health care in South China. Our previous study showed that FPH protected acute liver damage induced by alcohol. However, there is no study reporting FPH treating ulcerative colitis (UC). This study is designed to investigate whether FPH could inhibit UC and reveal its potential mechanism. The results showed that FPH significantly alleviated the UC disease symptoms including the body weight loss, disease activity index (DAI), stool consistency changing, rectal bleeding, and colon length loss of UC mice induced by dextran sulfate sodium (DSS) and reversed the influences of DSS on myeloperoxidase (MPO) and diamine oxidase activity (DAO). FPH suppressed UC via inhibiting the TLR4/MyD88/NF-*κ*B pathway and strengthened the gut barrier of mice via increasing the expressions of ZO-1 and occludin and enhancing the colonic antioxidative stress property by increasing the levels of T-SOD and GSH-Px and the expressions of NRF2, HO-1, and NQO1 and reducing MDA level and Keap1, p22-phox, and NOX2 expressions. Furthermore, FPH significantly inhibited SLD related to colitis by reducing the abnormal levels of the liver index, ALT, AST, and cytokines including TNF*α*, LPS, LBP, sCD14, and IL-18 in the livers, as well as decreasing the protein expressions of NLRP3, TNF*α*, LBP, CD14, TLR4, MyD88, NF-*κ*B, and p-NF-*κ*B, suggesting that FPH alleviated UC-related SLD via suppressing inflammation mediated by inhibiting the TLR4/MyD88/NF-*κ*B pathway. Our study firstly investigates the anticolitis pharmacological efficacy of FPH, suggesting that it can be enlarged to treat colitis and colitis-associated liver diseases in humans.

## 1. Introduction

Inflammatory bowel disease (IBD), including Crohn's disease (CD) and ulcerative colitis (UC), is a kind of chronic nonspecific inflammatory disease of the gut [1] and is the most risk for colorectal cancer (CRC) in developed countries [2]. Currently, the number of IBD patients in America is more than two million, and it is forecasted to be approaching four million by 2030 [3]. Epidemiological survey data showed that the prevalence rates were 505 per 100,000 in Europe, 248 per 100,000 in Canada, and 21.4 per 100,000 in the United States [4, 5]. The incidences of IBD in Asian countries are also sharply increasing in recent years, with a higher occurrence rate in women than men and shifting to adolescents, mainly due to the great changes in living environment and lifestyle, interacting with genetic factors [6, 7].

UC is a chronic inflammatory bowel disease with the characteristics of bloody diarrhea, abdominal cramping, and constant recurrence because of inflammatory cell infiltration [8]. Clinical and experimental evidence indicates that long-term inflammatory infiltration would destroy the intestinal mucosa barrier that cannot be restored [9]. It becomes a major clinical problem due to its repeated attacking and difficult curing. Currently, the treatments in the clinic are mainly including sulfasalazine, mesalazine, prednisone, azathioprine, methotrexate, antibodies, Janus kinase blockers, and stem cell-based therapy [10]. These therapeutics are not satisfying enough because they mainly relieve the disease symptoms but do not cure UC completely. Patients would relapse easily when the treatments terminate. Additionally, long-term taking of the drugs would lead to a series of side effects [11, 12]. Therefore, it is urgently demanded to develop a more safe and effective drug for UC therapy. Traditional herbal medicine is a good resource to discover UC therapeutic drugs on account of its long-time usage in clinics with the features of low toxicity, multicomponents, and multitargets.

The etiology of UC is still unclear. Multifactors including living and social environments, lifestyle, and genetic factors are mainly involved [2]. Oxidative stress plays a critical role in the onset of UC, during which excessive oxidation would destroy the tight junctions in the gut and promote the inflammatory factors to penetrate, therefore damaging the intestinal mucosa and aggravating the inflammatory response [13]. Furthermore, patients with UC are often accompanied by one or more extraintestinal symptoms (EIM), leading to multiple organ damage, such as liver injury [14]. During UC development, the intestinal mucosal barrier function would be impaired and intestinal permeability would be increased. Excessive growth of intestinal flora, together with their metabolites such as lipopolysaccharide (LPS), toxin, and reactive oxygen species (ROS), will enter into the liver through the portal vein system, by which the nonspecific immune system or proinflammatory signaling in the liver would be activated, producing a large number of inflammatory cytokines and chemokines, aggravating the inflammatory response, thus leading to the occurrence of liver injury or hepatitis [15]. Therefore, it is a good strategy to treat liver injury or hepatitis by suppressing UC and strengthening the gut barrier function. Studies reported that several traditional Chinese herbal medicines, such as *Curcuma longa* L., *Atractylodes macrocephala* Koidz., and *Dendrobium nobile* Lindl. possessing the UC therapeutic function, showed a good protective effect against liver damage [16–19].

*Ficus pandurata* Hance (FPH) is a Moraceae plant widely distributed in southern China, with high eatable and medicinal values. In Chinese traditional usage, FPH is considered to have the functions of clearing heat, promoting blood circulation, and protecting liver injury [20, 21]. In folk tradition, FPH is used to treat cough and make soup for daily health care. Our previous study had revealed that FPH could significantly alleviate acute alcohol-induced liver damage on mice [22]. However, there is no study reporting FPH treating UC and UC-related complications. Our present study is designed to investigate whether FPH could inhibit UC and UC-accompanied liver injury on colitis mice, as well as to uncover its underlying mechanism.

## 2. Materials and Methods

### 2.1. Reagents and Drug Preparation

Dextran sulfate sodium (DSS, MW: 36~50 kDa, S5036, 9011-18-1) was from MP Biomedicals Inc. (California, USA). Alanine transaminase (ALT, C010-2-1) and aspartate aminotransferase (AST, C009-2-1) assay kits were purchased from Nanjing Jiancheng Bioengineering Institute (Nanjing, China). MPO (#m1002070-2) and DAO (#m1002199-2) assay kits were purchased from Mlbio Biotechnology Co. (Shanghai, China). ELISA kits including MDA (417210607), GSH-Px (312210519), T-SOD (535210519), LPS (261210519), LBP (845210621), sCD14 (521210621), TNF*α* (569210610), and IL-18 (375210519) were purchased from Tianjin Anoric Biotechnology Co., Ltd. (Tianjin, China). Primary antibodies against ZO-1 (AF5145), occludin (DF7504), MyD88 (AF5195), HO1 (AF5393), NQO1 (DF6437), Keap1 (AF5266), NOX2 (DF6520), p22-phox (DF10099), LBP (DF4840), CD14 (DF13278), and *β*-actin (T0022) were obtained from Affinity Biosciences Ltd. (OH, USA). Primary antibodies against Nrf2 (A0674) and NLRP3 (A5652) were obtained from ABclonal Biotechnology (Wuhan, China). Primary antibodies against NF-*κ*B (8242s) and phospho-NF-*κ*B (3033T) were obtained from Cell Signaling Technology (Boston, USA). Primary antibodies against TLR4 (293072) and TNF*α* (YM3477) were obtained from Santa Cruz (California, USA) and ImmunoWay Biotechnology Company (Plano, USA), respectively.

The preparation of *Ficus pandurata* Hance extraction was performed according to our previous study [22].

### 2.2. UPLC/MS Q-TOF Analysis

Characteristic components of FPH were analyzed by Agilent LC/MS Q-TOF (USA, 1290-6545). The UPLC chromatographic conditions were ZORBAX Eclipse Plus C18 column (3.0 mm × 150 mm, 1.8 *μ*m), 30°C of column temperature, and mixed mobile phase (phase A: 0.1% of formic acid in water, mobile phase B: 100% acetonitrile) with a gradient elution program (5% B (0-2 min), 5% B → 35% B (2-20 min), 35% B → 50% B (20-24 min), 50% B → 40% B (24-25 min), 40% B → 95% B (25-30 min), and 95% B (30-35 min)) at 0.5 mL/min of flow rate and 0.5 *μ*L of injection volume. The mass spectrometer was the Agilent G6545 quadruple-time-of-flight spectrometer coupled with an Agilent Jet Stream Electrospray source (Agilent, CA, USA). The system was operated in positive or negative scan modes. The instrument parameters were 3500 kV (-) or 4000 V(+) of capillary voltage, N2 drying gas with 11 L/min of flow rate at 300°C temperature, 350°C of sheath gas temperature, 130 V of cataclastic voltage, 45 psi of nebulizer pressure, MS scan and *m*/*z* 100–1000, and 8 spectrums of the scan speed.

### 2.3. Experimental Design

Ten-week-old C57BL/6 mice (♂) were supplied by the Guangdong Laboratory Animal Center (Foshan, China). All the mice had access to food and water ad libitum and were kept under a condition with a 12 h light/dark cycle. The mice were randomly assigned to control, 2.5% DSS (model), 2.5% DSS+5-ASA (200 mg/kg, positive control), 2.5% DSS+FPH high dosage (FPH-H, 48 g/kg, calculated based on the quantity of crude material), and 2.5% DSS+FPH low dosage (FPH-L, 24 g/kg, calculated based on the quantity of crude material) groups. 5-ASA and FPH were given orally from day 1 to day 12, and the colitis mouse model was induced by 2.5% DSS (*W*/*V*) for 7 days (from day 6 to day 12). The mice were sacrificed 24 h after FPH treatment terminated.

### 2.4. Assessment of the Disease Activity Index (DAI)

The DAI scores were determined based on body weight change, rectal bleeding, and stool consistency as previously described [23]. The scoring system for DAI is shown in Table 1.

### 2.5. Sample Collection

After treatment, the mice were anesthetized to collect serum and then sacrificed for dissection. Briefly, blood was collected and serum was obtained by centrifugation at 4,000 rpm at 4°C for 10 min. The colon was removed for length measurement. Part of colon tissue was fixed in 4% paraformaldehyde (PFA) for hematoxylin and eosin (H&E) staining, and the rest of the part was stored at -80°C. The liver was also removed and weighted, with some tissues fixed for histopathological examination by H&E staining and other tissues stored at -80°C.

### 2.6. Enzyme-Linked Immunosorbent Assay (ELISA)

ELISA kits were obtained commercially, and biochemical analysis was performed according to the manufacturer's introduction. Briefly, colon and liver tissues were washed in cold phosphate buffer saline (PBS, 0.01 M, pH 7.4) and homogenized with 10 times of PBS (*V*/*W*). The samples were centrifuged at 5,000 g for 10 min at 4°C, and the supernatants were collected for biochemical analysis.

### 2.7. Western Blot Analysis

Colon tissues were lysed with RIPA lysis buffer supplemented with a protease inhibitor and phosphatase inhibitor (Beyotime, China) for 30 min on ice. The protein samples were collected after centrifugation at 15,000 rpm at 4°C for 10 min, and the protein concentrations were measured by using a bicinchoninic acid (BCA) protein assay kit (Beyotime, China). Equal amounts of protein (40 *μ*g) were loaded and separated on 8%~15% sodium dodecyl sulfate-polyacrylamide gels and transferred onto polyvinylidene difluoride (PVDF) membranes (Merck Millipore Ltd. IPVH00010, Darmstadt, Germany). The membranes were blocked with QuickBlock™ solution (Beyotime, P0252, Shanghai, China) at room temperature for 15 min, washed in PBST buffer, and incubated overnight at 4°C with primary antibodies of ZO-1, occludin, HO1, NQO1, Keap1, NRF2, NOX2, p22-phox, TLR4, MyD88, NF-*κ*B, phospho-NF-*κ*B, NLRP3, TNF*α*, LBP, CD14, and *β*-actin. After washing with PBST 3 times, the membranes were incubated with secondary antibodies conjugated with horseradish peroxidase (HRP) (1 : 10000) at room temperature for 1.5 h. The membrane blots were detected by using an enhanced chemiluminescence (ECL) kit. All gray analyses for protein blots were performed with ImageJ software.

### 2.8. Hematoxylin and Eosin (H&E) Staining

Histopathological examination was performed according to the reference with minor modification [24]. Briefly, colon and liver tissues were fixed in 4% PFA for 24 h, dehydrated by gradient ethanol, paraffin embedded, sectioned (~4 *μ*m thick), stained with H&E, and mounted with neutral gum. The morphological changes of tissues were observed under an optical microscope, and photos were taken (magnifications, 50x and 200x in colon tissues and 100x and 200x in liver tissues).

### 2.9. Immunohistochemistry (IHC) Study

IHC analysis was performed to examine the protein expressions of TLR4 and NF-*κ*B in colon tissues and NLRP3 in liver tissues of each group. Briefly, the paraffin-embedded samples were cut into sections (~4 *μ*m) and sealed with 3% H_2_O_2_ at room temperature to inactivate the enzyme, then boiled in 10 mM sodium citrate buffer (pH 6.0) for 10 minutes, and cooled at room temperature. The sections were blocked with normal goat serum and then incubated with anti-TLR4, anti-NF-*κ*B, and anti-NLRP3 primary antibodies (1 : 200) at 4°C overnight and corresponding secondary antibodies. The nucleus was stained with DAPI. The expressions of TLR4 and NF-*κ*B in colon tissues and the expression of NLRP3 in liver tissues were evaluated under an optical microscope, and photos were taken (magnification, 200x in colon and liver tissues).

### 2.10. Statistical Analysis

All data were expressed as mean ± standard error of the mean (SEM). The statistical differences between the two groups were compared by Student's *t*-test. Differences at *p* < 0.05 were considered statistically significant.

## 3. Results

### 3.1. Phytochemical Identification of FPH

**Figure 1:**
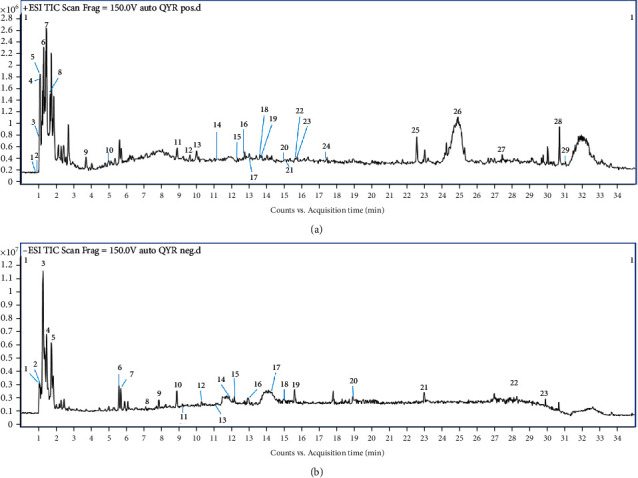
Identification of the compounds in FPH by UPLC/MS Q-TOF analysis. Data in (a) positive mode and (b) negative mode.

The components of FPH were analyzed by UPLC/MS Q-TOF. As shown in Figure 1, a total of 29 major compounds were identified by UPLC/MS Q-TOF in a positive mode (Figure 1(a)), including creatinine (peak 1), 2,3-dihydro-5,7-dihydroxy-2,8-dimethyl-6-(3-methyl-2-butenyl)-4H-1-benzopyran-4-one (peak 2), gamma-octalactone (peak 3), histamine (peak 4), sinapine (peak 5), nicotinic acid (peak 6), mimosine (peak 7), nicotinamide (peak 8), gentianaine (peak 9), tuliposide B (peak 10), cryptochlorogenic acid (peak 11), verbenalin (peak 12), tuberosine A (peak 13), bavachin (peak 14), quassimarin (peak 15), podolide (peak 16), loliolide (peak 17), icariside B9 (peak 18), miraxanthin III (peak 19), bruceine E (peak 20), angelol B (peak 21), tagitinin F (peak 22), erianin (peak 23), dihydrocapsaicin (peak 24), terpinyl acetate (peak 25), santenone alcohol (peak 26), gentianadine (peak 27), menthyl acetate (peak 28), and aristolophenanlactone I (peak 29). A total of 23 major compounds were identified in a negative mode (Figure 1(b)), including mesaconic acid (peak 1), pyroracemic acid (peak 2), trans-aconitic acid (peak 3), piceatannol (peak 4), succinic acid (peak 5), polydatin (peak 6), protocatechuic acid (peak 7), canthoside B (peak 8), 4-hydroxybenzoic acid (peak 9), geniposide (peak 10), justicidin B (peak 11), daphentin (peak 12), syringic acid (peak 13), methyl(2,4-dihydroxy-3-formyl-6-methoxy)phenylketone (peak 14), p-coumaric acid (peak 15), 7-hydroxycoumarin (peak 16), berberastine (peak 17), 4-hydroxybenzoic acid (peak 18), methyl-5,7-dihydroxy-2(Z)-octenoate (peak 19), 5,6,7-trimethoxycoumarin (peak 20), sanleng acid (peak 21), 6-gingerol (peak 22), and damascenine (peak 23). The information of all compounds that had been detected by UPLC/MS QTOF is shown in Supplementary Tables 1 and 2.

### 3.2. FPH Alleviated Ulcerative Colitis Induced by DSS on Mice

**Figure 2:**
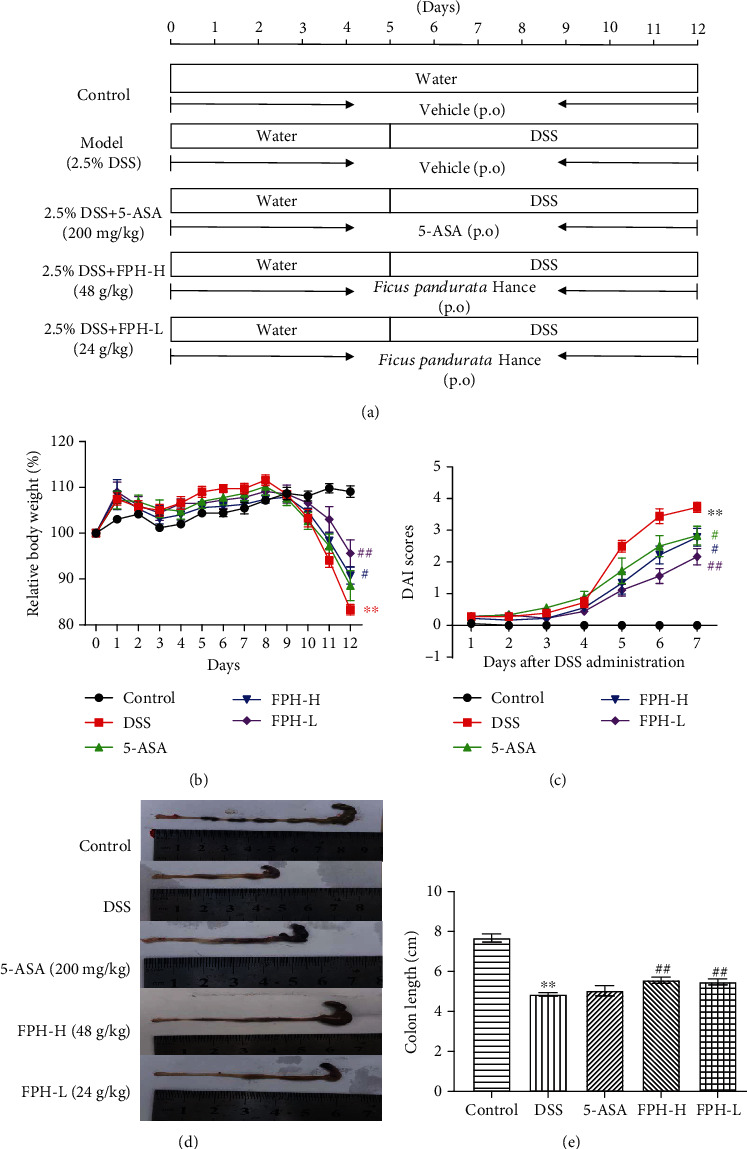
FPH alleviates DSS-induced acute colitis on mice. (a) Design for FPH in an anticolitis effect on the mouse model induced by DSS. Effect of FPH on body weight (b) and disease activity index (c) of colitis mice. (d) Representative photographs of the colon. (e) Effect of FPH on colon length of colitis mice. Results were expressed as mean ± SEM. ^∗∗^*p* < 0.01 vs. the control group; ^#^*p* < 0.05, ^##^*p* < 0.01 vs. the model group (DSS only).

The UC mouse model was established by taking 2.5% DSS orally to assess the therapeutic function of FPH against UC. The experiment design is shown in Figure 2(a). As a result, compared with the control group, the body weight loss, diarrhea, and rectal bleeding were severe in the DSS model group, which were the classical features of UC, while the body weight loss, diarrhea, and rectal bleeding were significantly delayed in FPH and 5-ASA treatment groups (Figure 2(b)). DAI scores in the model group were significantly higher than those in the control group, indicating that the UC model was established successfully. After FPH (24 g/kg and 48 g/kg, calculated based on the quantity of crude material) and 5-ASA (200 mg/kg) treatments, the high DAI scores induced by DSS were significantly reversed (Figure 2(c)), showing the good therapeutic efficacy of FPH and 5-ASA against UC. Furthermore, as shown in Figures 2(d) and 2(e), FPH and 5-ASA could significantly lengthen the colon of UC mice. These data demonstrated that FPH could be a good candidate for UC therapy.

### 3.3. FPH Inhibited UC via Suppressing the TLR4/MyD88/NF-*κ*B Pathway

**Figure 3:**
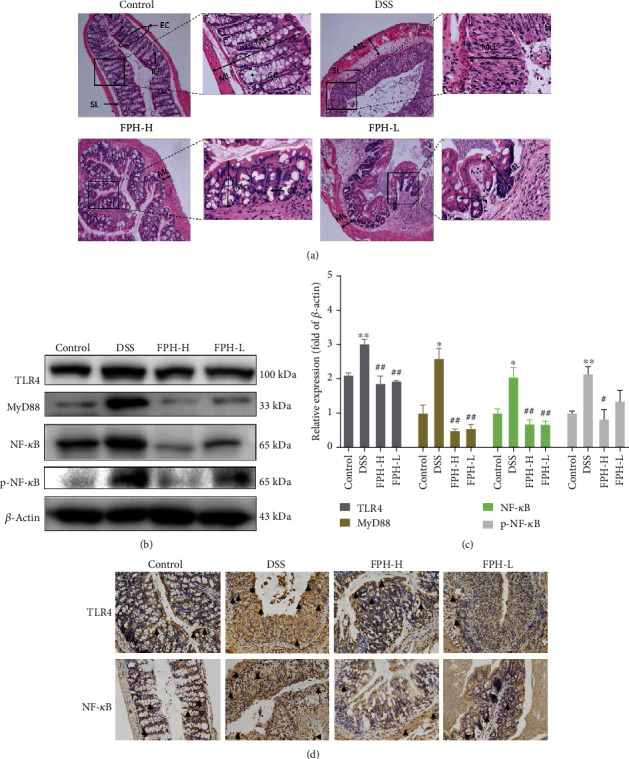
FPH suppresses DSS-induced colitis in mice by inhibiting the TLR4/MyD88/NF-*κ*B signaling pathway. (a) Histological analysis for the colon by H&E staining (EC: epithelial cell; IG: intestinal glands; SL: submucous layer; ML: muscular layer; McL: mucous layer; GC: goblet cell). The protein expressions of TLR4, MyD88, NF-*κ*B, and phospho-NF-*κ*B in colonic tissues were determined by Western blot (b) and quantitative analysis for Western blot results by normalizing to *β*-actin (c). (d) The protein expression levels of TLR4 and NF-*κ*B in colonic tissues were detected by IHC. The black arrows were showing the positive cells. Results were expressed as mean ± SEM. ^∗^*p* < 0.05, ^∗∗^*p* < 0.01 vs. the control group; ^#^*p* < 0.05, ^##^*p* < 0.01 vs. the model group (DSS only).

The effect of FPH on histopathological morphology of colon tissues in UC mice was determined via H&E staining. As shown by the arrow in Figure 3(a), in the control group, there are many columnar cells in the intestinal mucosal layer, with the shapes being regular, arranged, and full of goblet cells. After DSS induction, the histopathological morphology changed remarkably, including that the mucosal layer was destroyed, goblet cells were almost invisible, and both the muscular layer and mucosal layer were widened, indicating that the intestinal permeability was significantly increased. In addition, the inflammatory infiltration in the mucosal layer was enhanced in the DSS induction group. However, after FPH treatment, the goblet cells in the intestinal mucosa were restored and the columnar cells were arranged regularly, showing that the intestinal permeability and inflammation were ameliorated. The TLR4/MyD88/NF-*κ*B signaling pathway plays a critical role in the development of UC [25]. In this study, Western blot and IHC assays were used to evaluate the effect of FPH on the TLR4/MyD88/NF-*κ*B signaling pathway. As shown by the results in Figures 3(b)–3(d), DSS significantly increased the expressions of TLR4, MyD88, NF-*κ*B, and phospho-NF-*κ*B in the colon tissues (*p* < 0.05 vs. control group), while FPH strongly suppressed the high levels of TLR4, MyD88, NF-*κ*B, and phospho-NF-*κ*B induced by DSS in the colon (*p* < 0.01 vs. model group). All of the results indicated that FPH could inhibit UC via suppressing the TLR4/MyD88/NF-*κ*B signaling pathway.

### 3.4. FPH Enhanced the Intestinal Barrier of UC Mice

**Figure 4:**
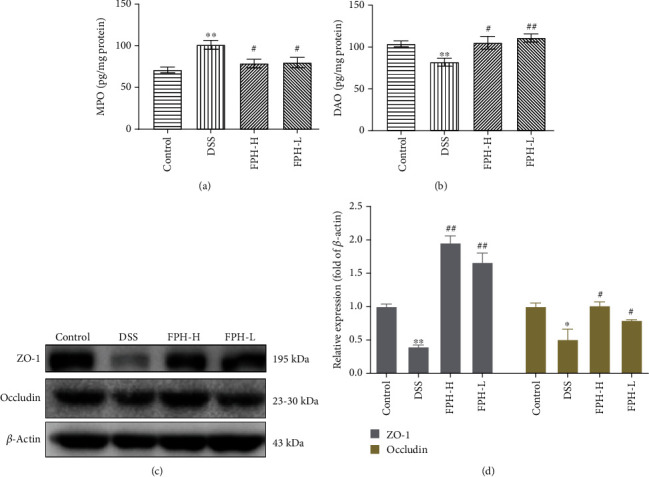
Effect of FPH on the intestinal barrier of IBD mice. (a) Effect on MPO concentration of the colonic homogenate. (b) Effect on DAO concentration of the colonic homogenate. The expression levels of tight junction proteins ZO1 and occludin in colon tissues were evaluated by Western blot (c) and quantitative analysis for Western blot results by normalizing to *β*-actin (d). Results were expressed as mean ± SEM. ^∗^*p* < 0.05, ^∗∗^*p* < 0.01 vs. the control group; ^#^*p* < 0.05, ^##^*p* < 0.01 vs. the model group (DSS only).

Myeloperoxidase (MPO), a glycoprotein promoting a series of peroxidative stress responses when neutrophils are stimulated, is a key marker reflecting the development of intestinal inflammation [26]. Diamine oxidase reflects intestinal mucosal injury and intestinal barrier [27]. As shown by the results in Figures 4(a) and 4(b), DSS induced a high level of MPO and a low level of DAO in the colon, compared with the control group. After FPH treatment, the influences of DSS on MPO and DAO were significantly reversed (*p* < 0.01 vs. model group). Furthermore, the effect of FPH on tight junction (TJ) protein expressions including ZO-1 and occludin in the colon tissues was investigated via the Western blot assay. The results showed that FPH could significantly increase the protein expressions of ZO-1 and occludin in colon tissues of the DSS-induced colitis mouse model (Figures 4(c) and 4(d)), indicating the strengthening function of FPH on the gut barrier, which was consistent with the H&E staining result.

### 3.5. FPH Enhances Colonic Antioxidation in the Colitis Mouse Model

**Figure 5:**
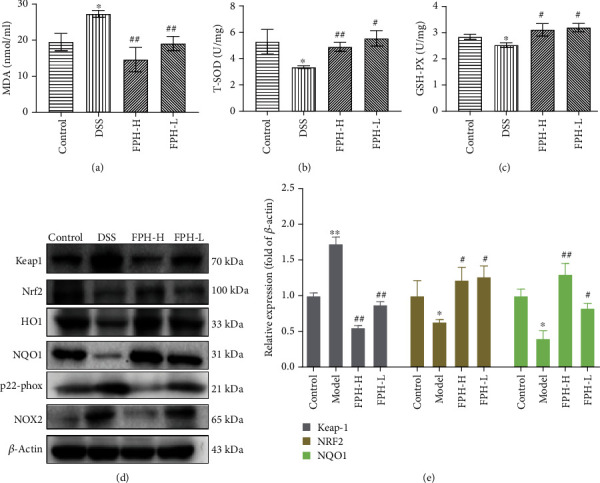
FPH enhances the colonic antioxidation in the UC mice induced by DSS. Effect of FPH on the levels of MDA (a), T-SOD (b), and GSH-Px (c) in the colonic homogenate detected by ELISA. (d) Effect of FPH on the protein expressions of Keap1, Nrf2, HO1, NQO1, p22-phox, and NOX2 in colon tissues detected by Western blot. Results were expressed as mean ± SEM. ^∗^*p* < 0.05, ^∗∗^*p* < 0.01 vs. the control group; ^#^*p* < 0.05, ^##^*p* < 0.01 vs. the model group (DSS only).

Oxidative stress has been recognized as an important mechanism underlying the pathophysiology of IBD [28]. Hence, targeting oxidative stress is a good strategy for IBD therapy. In our present study, we investigated the influence of FPH on oxidative stress of the colon in DSS-induced colitis mice via ELISA and Western blot assays. As shown in Figures 5(a)–5(c), the levels of total superoxide dismutase (T-SOD) and glutathione peroxidase (GSH-Px), two important antioxidation parameters, were 5significantly decreased (*p* < 0.05 vs. control group), and malondialdehyde (MDA) was significantly increased after DSS induction (*p* < 0.01 vs. control group). After FPH treatment, the influences of DSS on T-SOD, GSH-Px, and MDA were significantly reversed (*p* < 0.05 vs. model group). Additionally, the effects of FPH on oxidative stress-related protein expressions were assessed, including Keap1, Nrf2, HO1, NOX2, p22-phox, and NQO1. The results in Figures 5(d) showed that DSS markedly upregulated the expressions of Keap1, NOX2, and p22-phox, three important proteins producing ROS, and downregulated Nrf2 and its downstream factor heme oxygenase-1 (HO1), as well as quinone oxidoreductase 1 (NQO1), a cytosolic antioxidant flavoprotein catalyzing the oxidation of NAD(P)H to NAD(P)+ [29]. However, after FPH treatment, the levels of Keap1, NOX2, and p22-phox in colon tissues were strongly reduced, and the levels of Nrf2, HO1, and NQO1 were remarkably increased, indicating the good regulation of FPH on oxidative stress of the colon. Altogether, these data demonstrated that FPH could alleviate ulcerative colitis in mice via strengthening the gut barrier mediated by enhancing the antioxidation activity of the colon.

### 3.6. FPH Inhibited Colitis-Associated Secondary Liver Damage of Mice

**Figure 6:**
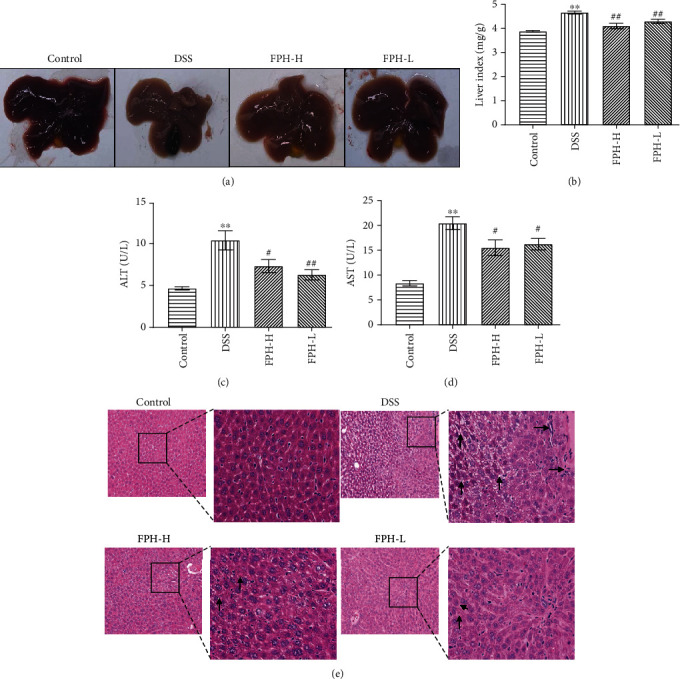
FPH alleviates secondary liver damage in DSS-induced acute colitis in mice. (a) Representative photographs of liver tissue after FPH treatment. Effect of FPH on the liver organ index (b) and levels of ALT (c) and AST (d) in serum of IBD mice. (e) Histological analysis for liver tissues by H&E staining. The black arrows were indicating the cells with hepatic steatosis in liver tissues of UC mice. Results were expressed as mean ± SEM. ^∗^*p* < 0.05, ^∗∗^*p* < 0.01 vs. the control group; ^#^*p* < 0.05, ^##^*p* < 0.01 vs. the model group (DSS only).

Secondary liver injury is commonly accompanied by IBD in the clinic and would probably be aggravated, leading to chronic hepatitis or hepatic fibrosis [30, 31]. In our study, the protective effect of FPH on liver injury was evaluated in the DSS-induced UC mouse model. As shown in Figure 6(a), the appearance of liver tissue changed obviously after DSS induction, while FPH could significantly reverse the liver appearance change induced by DSS. The liver index data showed that FPH could retard the influence of DSS on the liver index of mice (Figure 6(b)). Furthermore, the serum levels of ALT and AST (Figures 6(c) and 6(d)), two biochemical parameters reflecting liver injury, were also significantly reversed after FPH treatment (*p* < 0.05 vs. model group). Additionally, the H&E staining assay showed that the liver tissues in the DSS group mice had typical features of liver injury, including disorderly arranged liver cells, enlarged vacuoles, unclear cell edge, broken cell membranes, and visibly appeared necrotic areas. After FPH treatment, the classical features of pathological changes in liver tissues were remarkably ameliorated (Figure 6(e)). In all, these data indicated that FPH could inhibit ulcerative colitis-associated secondary liver damage in mice.

### 3.7. FPH Protected Secondary Liver Injury of UC Mice via Regulating the Gut-Liver Axis

**Figure 7:**
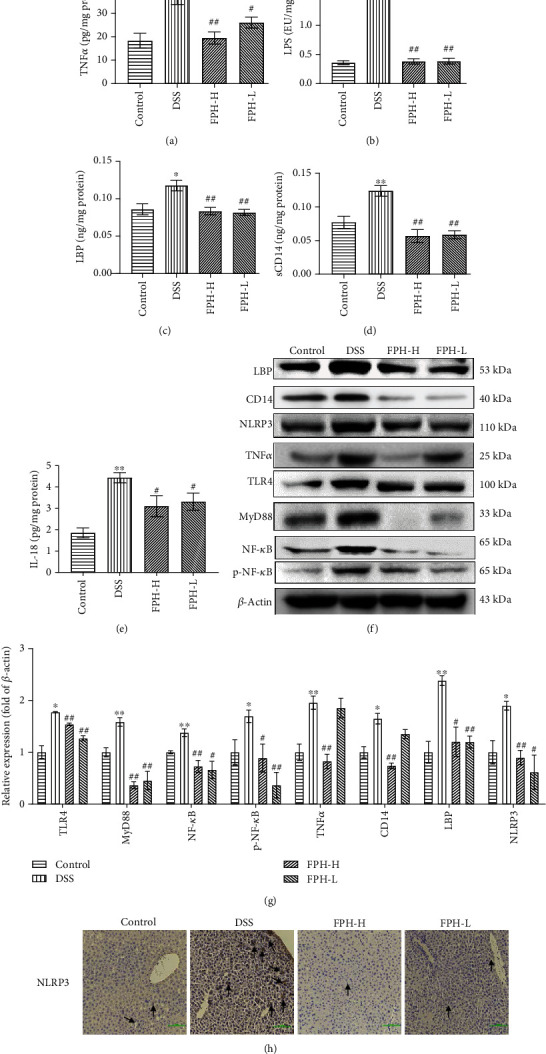
FPH alleviates secondary liver injury in colitis mice via suppressing inflammation and the LPS/LBP/CD14/TLR4/NF-*κ*B signaling axis. Effect of FPH on the levels of TNF*α* (a), LPS (b), LBP (c), sCD14 (d), and IL-18 (e) in the liver tissues of UC mice determined by ELISA. The protein expressions of LBP, CD14, NLRP3, TNF*α*, TLR4, MyD88, NF-*κ*B, and p-NF-*κ*B in the liver tissues were evaluated by Western blot (f) and quantitative analysis for Western blot results by normalizing to *β*-actin (g). (h) The protein expression of NLRP3 in the liver tissues of UC mice was detected by IHC. Results were expressed as mean ± SEM. ^∗^*p* < 0.05, ^∗∗^*p* < 0.01 vs. the control group; ^#^*p* < 0.05, ^##^*p* < 0.01 vs. the model group (DSS only).

This study had shown that FPH alleviated UC by suppressing the TLR4/MyD88/NF-*κ*B pathway and inhibited UC-associated secondary liver damage in mice. It had been reported that the gut-liver axis played a key role in the pathogenesis of liver diseases and gut-derived lipopolysaccharide (LPS) played a central role in the induction of organ injury, inflammation, and fibrosis of the liver through the portal circulation [32]. Hence, we speculated that the underlying mechanism of FPH protecting secondary liver injury of UC mice was probably related to the regulation of the gut-liver axis. Therefore, the levels of IL-18, LPS, and LPS-related signaling factors in livers, such as soluble CD14 (sCD14), LPS-binding protein (LBP), and TNF*α*, were determined via ELISA and Western blot assays. As we speculated, the data in Figures 7(a)–7(e) showed that DSS strongly increased the levels of TNF*α*, LPS, LBP, sCD14, and IL-18 in the liver tissues (*p* < 0.05 vs. control group), while FPH significantly reversed the effect of DSS on mice (*p* < 0.05 vs. model group). Meanwhile, the Western blot assay showed that DSS increased the expressions of LBP, CD14, TNF*α*, and NLRP3 in the liver tissues (*p* < 0.05 vs. control group), while FPH significantly decreased the expressions of these proteins in the liver tissues (*p* < 0.05 vs. DSS group). The TLR4/MyD88/NF-*κ*B pathway plays a critical role in the inflammatory response and liver injury. Hence, we also detected the expressions of the TLR4/MyD88/NF-*κ*B signaling pathway in the liver tissues of UC mice. As a result, DSS significantly increased the expressions of TLR4, MyD88, NF-*κ*B, and phospho-NF-*κ*B in the liver tissues, while FPH strongly reversed the effect (Figures 7(f) and 7(g)), indicating the excellent alleviated effect of FPH against inflammatory response and liver injury. The IHC result also validated that FPH obviously reduced the expression of NLRP3, which was consistent with the Western blot data (Figure 7(h)).

All in all, these results showed that FPH could alleviate ulcerative colitis and ulcerative colitis-associated secondary liver damage by strengthening the gut barrier of mice via enhancing the antioxidation activity of the colon.

## 4. Discussion

Ulcerative colitis (UC) is a chronic, nonspecific inflammatory bowel disease caused by an interaction of genetic background with environmental factors. The incidence of UC is rising rapidly, with characteristics of the chronic disease course, repeated attacks, gradual aggravation, and life-threatening in severe cases [33]. At present, the clinical drugs for UC treatment are not so satisfying owing to their low efficacy and obvious side effects for long time usage. Herbal medicine has the characteristics of multitargets, multisignaling pathways, reducing recurrence rate, and treating complications against IBD. According to the basic theory of traditional herbal medicine in China, IBD belongs to damp-heat, static blood, and poison accumulated in the colon tract recorded in Yellow Emperor's Inner Canon. Therefore, herbal medicines or prescriptions with the functions of heat-clearing, detoxication, activating blood, and eliminating dampness would have good therapeutic efficacy against IBD, such as *Trametes robiniophila* Murr., *Curcuma longa* L., and *Plantago depressa* Willd. [17, 34, 35]. *Ficus pandurata* Hance, mainly containing the chemical constituents of triterpenes, flavonoids, coumarins, sterols, and so on [36, 37], had been recorded to possess the functions of clearing heat, detoxification, and anti-inflammation in the traditional Chinese medicine documentation [22]. Therefore, *Ficus pandurata* Hance would be probably used for IBD therapy according to the basic theory of traditional China herbal medicine, which has been validated in our experimental study.

Inflammation is the key characteristic of IBD. DSS, a low-molecular-weight sulfated polysaccharide, is a common chemical used to induce IBD through increasing the secretion of proinflammatory cytokines, affecting the distribution of tight junction proteins and even destroying the intestinal epithelial cell (IEC) structure, which would lead to innate immune cell response [38]. Activated immune cells would release inflammatory cytokines to regulate different kinds of cells [39]; for instance, neutrophil and macrophage would induce T helper cells to produce IL-6 and TNF*α* and mononuclear phagocytes and intestinal epithelial cells produced IL-1*β* and IL-18, aggravating inflammation [40–42], while dendritic cells would activate T regulatory cells to secrete IL-10 and TGF*β*, playing a protective role [43–45]. During inflammation evolution, the proinflammatory factors continuously stimulate the immune response, promoting the body to develop into chronic inflammation, which is a major pathological cause of many serious diseases including cancer, diabetes, organ failure, and so on [46]. It is reported that IBD is prone to colonic carcinogenesis, and the incidence of colitis-related colorectal cancer is 4~10 times higher than that of sporadic colorectal cancer [47, 48]. Hence, inhibiting the acute inflammatory response and retarding chronic inflammation evolution can prevent other diseases such as colorectal cancer from occurring.

Increasing evidence shows that oxidative stress can promote IBD [13, 49]. In patients with ulcerative colitis, the reactive oxygen metabolites of the intestinal mucosa increase and would break the balance of oxidation and antioxidation [50]. Studies have shown that under the action of reductive coenzymes and magnesium oxide in the cell membrane, a large number of superoxide anions, hydroxyl radical, hydrogen peroxide, and lipid peroxides are produced through cellular respiration and further induce chemotaxis of neutrophils, leading to inflammatory infiltration of colon tissue [51]. NOX1 and NOX2, two important family members of NOXs, are the main catalytic enzymes that catalyze the generation of oxygen free radicals in the intestinal mucosa [52]. When IBD happens, the expressions of NOX1 and NOX2 abnormally elevate, the generation of oxygen free radicals including MDA increases, and the levels of GSH and SOD decrease, leading to oxidative stress injury on the intestinal mucosa [53]. Excessive oxygen metabolites would promote inflammation and harm to DNA, proteins, and lipids, which are destroying the structural integrity of cells [54]. On the other side, Nrf2 plays a key role in the body's response to oxidative stress by regulating the expressions of antioxidant genes. Once being activated, Nrf2 enters the nucleus and combines with the antioxidant response element (ARE) to regulate the antioxidant enzymes and phase II detoxification enzymes such as heme oxygenase 1 (HO-1) and benzoquinone oxidoreductase (NQO1) [55–57]. Therefore, targeting oxidative stress is an important mechanism for IBD treatment. In our study, we assessed the expressions of Keap1, Nrf2, HO1, NQO1, NOX2, and p22-phox in the colon tissues via the Western blot assay and the levels of MDA, GSH-Px, and T-SOD, three important markers of oxidative stress, via ELISA. Our results showed that FPH significantly increased the levels of SOD and GSH and promoted the expressions of Nrf2, HO1, and NQO1, while it inhibited the expressions of Keap1 and NOX2 and reduced MDA level, indicating that FPH could strongly suppress the oxidative stress of the colon in IBD mice.

Lipopolysaccharide is an endotoxin derived from gram-negative bacteria, penetrating the gut mucosa only in trace amounts in normal physiological status, while in pathological status, it can increase the levels of proinflammatory cytokines by stimulating toll-like receptors such as TLR4 [25, 58]. Once TLR4 is activated by LPS, the cell signal transduction would be triggered, and the downstream target genes MyD88 and NF-*κ*B would be activated, thereby promoting the development of inflammation, such as the formation of pro-IL-18 [59]. Meanwhile, the NLRP3 inflammasome and oxygen free radicals promoted the maturation of pro-IL-1*β* and pro-IL-18 and then augmented the production of proinflammatory cytokines [60]. Studies found that TLR4 and its downstream factors are significantly enhanced in IBD, and the LPS/TLR4 pathway plays an important role in the occurrence and development of organ damage response and vicious circle [61–63]. The intestinal mucosa barrier, composed of intestinal epithelial cells (IECs) and tight junction proteins, is an important defense system maintaining the integrity of the barrier function and intestinal homeostasis by isolating harmful substances in the intestine [64]. Tight junction proteins mainly consist of zonula, occludin, and claudins, which regulate the permeability of the intestinal barrier and maintain the polarity of epithelial cells [65, 66]. Studies showed that IL-18 would directly inhibit the goblet cell maturation and then do harm to the defense function of the intestinal mucosal layer [67]. Continuous inflammatory response induced by LPS would destroy the morphology of the intestinal epithelium and aggravate the mucosal damage via decreasing the expression of intestinal tight junction proteins and increasing the intestinal permeability, by which more inflammatory factors would be gathered and infiltrate the intestine, forming a vicious circle. Han et al. found that LPS influenced the microbiota and energy metabolism of the gut-liver axis in the colitis model [68]. Therefore, suppressing the LPS-mediated TLR4/MyD88/NF-*κ*B pathway is regarded as an important strategy to treat IBD, which has been validated in our study.

Extraintestinal liver damage is one of the most common complications in severe IBD. Trivedi and Adams reported that colitis can cause liver injury, and the degree of liver injury is positively correlated with the severity of colitis [69]. When IBD occurs, the intestinal permeability is enhanced, and entheogenic bacterial metabolites, such as LPS, toxins, ROS, and short-chain fatty acid (SCFA), enter into the liver through the portal circulation. Hence, the levels of LPS or other endotoxins synthesized by gram-negative bacteria would be increased in the liver, inducing hepatocholangeitis and liver injury [68]. Under normal physiological status, LPS can be cleared in the liver to maintain the control of immune homeostasis, while under pathological conditions such as liver damage, hepatic fibrosis, or cirrhosis, LPS clearance from the circulation is decreased, and excessive LPS would activate innate immune cells, including Kupffer cells, leading to promoting various proinflammatory cytokines, chemokines, and other factors to release and reinforce the inflammatory response [70]. The LPS/TLR4 pathway plays a key role in triggering liver injury. It is reported that LPS binds to LPS-binding protein (LBP) and transfers to the cluster of differentiation 14 (CD14) and then binds to TLR4/myeloid differentiation factor-2 (MD-2) complex. This signal can be passed through MyD88-dependent intracellular pathways, which would activate downstream transcription factors and proinflammatory cytokines such as tumor necrosis factor *α* (TNF*α*) [71, 72]. In this study, we speculated that FPH could probably protect secondary liver injury induced by DSS through regulating the gut-liver axis mediated by suppressing LPS/LBP/CD14 signaling. As it was expected, the results showed that FPH obviously alleviated the secondary liver injury of UC mice including alleviating the steatosis and mitigating inflammation of the liver and significantly reduced the levels of ALT and AST in serum and IL-18, NLRP3, LPS, LBP, sCD14, and TNF*α* in liver tissues.

In summary, this study demonstrated that FPH not only could inhibit ulcerative colitis via suppressing the LPS/TLR4/MyD88/NF-*κ*B pathway and strengthen the gut barrier via enhancing the antioxidation of the colon but also could protect against secondary liver injury accompanied by ulcerative colitis.

## 5. Conclusion

In this study, the suppression of *Ficus pandurata* Hance against IBD on mice and the protective effect on secondary liver injury were investigated, as well as the underlying mechanism. The data showed that *Ficus pandurata* Hance had a good alleviated effect on IBD induced by DSS and a protective effect on secondary liver injury accompanied by IBD, mainly through inhibiting the inflammatory pathway LPS/TLR4/MyD88/NF-*κ*B and enhancing the gut barrier through suppressing oxidative stress of the colon. Our study for the first time provides experimental evidence for the therapeutic effect of *Ficus pandurata* Hance against IBD and secondary liver injury, which strongly suggests its application in IBD therapy for humans in the future.

## Figures and Tables

**Table 1:** Scoring system for DAI.

Score	Weight loss	Stool consistency	Blood stool
0	None	Normal	None
1	1~5%	Soft stool	Slight occult blood
2	5~10%	Paste stool	Occult blood
3	10~15%	Loose stool	Bleeding
4	>15%	Diarrhea	Gross bleeding

## Data Availability

The datasets used to support the current study are available from the corresponding author upon reasonable request.

## Abbreviations

IBD: Inflammation bowel disease
FPH: *Ficus pandurata* Hance
UC: Ulcerative colitis
CD: Crohn's disease
CRC: Colorectal cancer
DAI: Disease activity index
DSS: Dextran sulfate sodium
EIM: Extraintestinal symptoms
SCFA: Short-chain fatty acid
ROS: Reactive oxygen species
IECs: Intestinal epithelial cells
TJ: Tight junction
MPO: Myeloperoxidase
DAO: Diamine oxidase
TLR4: Toll-like receptor 4
MyD88: Myeloid differentiation factor 88
NF-*κ*B: Nuclear factor-*κ*-gene binding
MD2: Myeloid differentiation factor-2
T-SOD: Total superoxide dismutase
GSH-Px: Glutathione peroxidase
MDA: Malondialdehyde
Keap1: Kelch-like ECH-associated protein 1
Nrf2: Nuclear factor E2-related factor 2
HO-1: Heme oxygenase 1
NQO1: NADH quinone oxidoreductase 1
LPS: Lipopolysaccharide
LBP: LPS-binding protein
ALT: Alanine aminotransferase
AST: Aspartate aminotransferase
sCD14: Soluble CD14
TNF*α*: Tumor necrosis factor *α*
NOX2: NADPH oxidase 2.

## Supplementary Materials

Supplementary 1 Table S1: identified compounds of FPH by UPLC/MS QTOF in the positive ion mode.

Supplementary 2 Table S2: identified compounds of FPH by UPLC/MS QTOF in the negative ion mode.
