# Supplementary material for: Ficus pandurata Hance Inhibits Ulcerative Colitis and Colitis-Associated Secondary Liver Damage of Mice by Enhancing Antioxidation Activity
Source: Oxid Med Cell Longev. 2021 Dec 18;2021:2617881. doi: 10.1155/2021/2617881 (PMC8710911; doi:10.1155/2021/2617881)
Supplement: Supplementary 1 — Table S1: identified compounds of FPH by UPLC/MS QTOF in the positive ion mode. [file 2617881.f1.docx]

SUPPLEMENTARY TABLE 1. Identified compounds of FPH by UPLC/MS QTOF in the positive ion mode.

| Peak | Name | Mass | RT | Height | Formula |
| --- | --- | --- | --- | --- | --- |
| 1 | Creatinine | 113.0584 | 1.252 | 9111 | C4 H7 N3 O |
| 2 | 2,3-Dihydro-5,7-dihydroxy- 2,8-dimethyl-6-(3-methyl-2- butenyl)-4H-1-benzopyran- 4-one | 276.1366 | 1.318 | 10894 | C16 H20 O4 |
| 3 | gamma-Octalactone | 142.099 | 1.403 | 32516 | C8 H14 O2 |
| 4 | Histamine | 111.0792 | 1.424 | 94075 | C5 H9 N3 |
| 5 | Sinapine | 327.1672 | 1.59 | 56071 | C16 H25 N O6 |
| 6 | Nicotinic acid | 123.0316 | 1.658 | 34940 | C6 H5 N O2 |
| 7 | Mimosine | 198.0634 | 1.807 | 93458 | C8 H10 N2 O4 |
| 8 | Nicotinamide | 122.0476 | 1.883 | 22820 | C6 H6 N2 O |
| 9 | Gentianaine | 141.0421 | 2.96 | 27849 | C6 H7 N O3 |
| 10 | Tuliposide B | 294.0941 | 5.616 | 20506 | C11 H18 O9 |
| 11 | Cryptochlorogenic acid | 354.0937 | 8.504 | 5622 | C16 H18 O9 |
| 12 | Verbenalin | 388.1356 | 8.904 | 20951 | C17 H24 O10 |
| 13 | Tuberosine A | 343.1409 | 9.07 | 5269 | C19 H21 N O5 |
| 14 | Bavachin | 324.1316 | 10.346 | 6586 | C20 H20 O4 |
| 15 | Quassimarin | 522.2085 | 12.735 | 13072 | C26 H34 O11 |
| 16 | Podolide | 330.1456 | 13.627 | 6015 | C19 H22 O5 |
| 17 | Loliolide | 196.1096 | 14.174 | 16500 | C11 H16 O3 |
| 18 | Icariside B9 | 372.2137 | 15.031 | 5501 | C19 H32 O7 |
| 19 | Miraxanthin III | 330.1209 | 15.333 | 8536 | C17 H18 N2 O5 |
| 20 | Bruceine E | 412.174 | 16.369 | 8295 | C20 H28 O9 |
| 21 | Angelol B | 376.151 | 16.47 | 6098 | C20 H24 O7 |
| 22 | Tagitinin F | 348.1581 | 17.252 | 5753 | C19 H24 O6 |
| 23 | Erianin | 318.1479 | 17.537 | 8267 | C18 H22 O5 |
| 24 | Dihydrocapsaicin | 307.2132 | 18.565 | 7935 | C18 H29 N O3 |
| 25 | Terpinyl acetate | 196.1455 | 22.24 | 7837 | C12 H20 O2 |
| 26 | Santenone alcohol | 140.1196 | 24.556 | 50874 | C9 H16 O |
| 27 | Gentianadine | 149.0474 | 26.002 | 11165 | C8 H7 N O2 |
| 28 | Menthyl acetate | 198.1615 | 28.486 | 8332 | C12 H22 O2 |
| 29 | Aristolophenanlactone I | 310.0474 | 29.322 | 6597 | C17 H10 O6 |
