# Supplementary material for: Ficus pandurata Hance Inhibits Ulcerative Colitis and Colitis-Associated Secondary Liver Damage of Mice by Enhancing Antioxidation Activity
Source: Oxid Med Cell Longev. 2021 Dec 18;2021:2617881. doi: 10.1155/2021/2617881 (PMC8710911; doi:10.1155/2021/2617881)
Supplement: Supplementary 2 — Table S2: identified compounds of FPH by UPLC/MS QTOF in the negative ion mode. [file 2617881.f2.docx]

SUPPLEMENTARY TABLE 2. Identified compounds of FPH by UPLC/MS QTOF in the negative ion mode.

| Peak | Name | Mass | RT | Height | Formula |
| --- | --- | --- | --- | --- | --- |
| 1 | Mesaconic acid | 130.0262 | 1.41 | 109771 | C5 H6 O4 |
| 2 | Pyroracemic acid | 88.016 | 1.469 | 205186 | C3 H4 O3 |
| 3 | trans-Aconitic acid | 174.0163 | 1.535 | 198130 | C6 H6 O6 |
| 4 | Piceatannol | 244.0724 | 1.897 | 90393 | C14 H12 O4 |
| 5 | Succinic acid | 118.0261 | 2.313 | 205882 | C4 H6 O4 |
| 6 | Polydatin | 390.1296 | 5.609 | 97483 | C20 H22 O8 |
| 7 | Protocatechuic acid | 154.0271 | 5.908 | 195690 | C7 H6 O4 |
| 8 | Canthoside B | 464.1548 | 7.121 | 101928 | C19 H28 O13 |
| 9 | 4-Hydroxybenzoic acid | 138.0312 | 7.851 | 226377 | C7 H6 O3 |
| 10 | Geniposide | 388.1379 | 8.893 | 145037 | C17 H24 O10 |
| 11 | Justicidin B | 364.093 | 8.897 | 88287 | C21 H16 O6 |
| 12 | Daphentin | 178.0287 | 9.489 | 147707 | C9 H6 O4 |
| 13 | Syringic acid | 198.0559 | 10.117 | 80371 | C9 H10 O5 |
| 14 | Methyl(2,4-dihydroxy-3-formyl-6- methoxy)phenylketone | 210.0529 | 11.781 | 185246 | C10 H10 O5 |
| 15 | p-Coumaric acid | 164.0494 | 12.17 | 175072 | C9 H8 O3 |
| 16 | 7-Hydroxycoumarin | 162.0314 | 12.91 | 216226 | C9 H6 O3 |
| 17 | Berberastine | 352.1178 | 13.885 | 82668 | C20 H18 N O5 |
| 18 | 4-Hydroxybenzoic acid | 138.0333 | 14.996 | 99807 | C7 H6 O3 |
| 19 | Methyl-5,7-dihydroxy- 2(Z)-octenoate | 188.1046 | 15.587 | 194648 | C9 H16 O4 |
| 20 | 5,6,7-Trimethoxycoumarin | 236.0688 | 18.921 | 142149 | C12 H12 O5 |
| 21 | Sanleng acid | 330.2412 | 22.96 | 253843 | C18 H34 O5 |
| 22 | 6-Gingerol | 294.1839 | 27.052 | 192085 | C17 H26 O4 |
| 23 | Damascenine | 195.0899 | 28.27 | 174225 | C10 H13 N O3 |
